# Perceptions and practices of self-management among adult patients with gout at a primary care clinic: A qualitative study

**DOI:** 10.51866/oa.428

**Published:** 2023-12-18

**Authors:** Suhashini Sivasegaran, Nik Sherina Haidi Hanafi

**Affiliations:** 1 MD, MMed, Klinik Kesihatan Sandakan, K.M 3.2, Jln Utara Beg Berkunci No. 4, Sandakan, Sabah, Malaysia. Email: shassni26@gmail.com; 2 MBBS, MFamMed, PhD, Department of Primary Care Medicine, Faculty of Medicine, University of Malaya, Kuala Lumpur, Malaysia.

**Keywords:** Perception, Self-management, Practices, Gout

## Abstract

**Introduction:**

Gout is a chronic disease commonly associated with other comorbidities. Patients’ perceived quality of life empowers them in managing their health. Self-management is imparted as part of management among patients with chronic disease. This study aimed to explore the perceptions and practices of self-management among patients with gout from different ethnic groups in Malaysia.

**Methods:**

A qualitative study was conducted among Malay, Chinese and Indian patients with gout via semi-structured in-depth interviews at the primary care clinic of University Malaya Medical Centre in either English or Malay language. All participants had a gout duration of more than 6 months and were either taking urate-lowering drugs or not using them at all.

**Results:**

A total of 20 participants were successfully recruited for the study. Among the participants, 18 were men, while two were women. Further, nine were Malays; six, Chinese; and four, Indians. The age ranged from 29 to 81 years, while the gout duration ranged from 1 to 30 years. From the interviews, three themes emerged: experiences with gout, types of self-management of gout and factors influencing self-management of gout.

**Conclusion:**

Diet control is the main self-management practice of patients with gout. Traditional medicine practices include natural methods such as consumption of different types of vegetable juices, pineapple and papaya. Each ethnicity has its own unique beliefs and food cultures. By understanding the self-management practices of patients from diverse ethnic backgrounds, healthcare practitioners can tailor the treatment of gout to individual needs.

## Introduction

Gout is a common inflammatory arthritis caused by defective metabolism of uric acid and is predominantly managed in primary care settings.^1,2^ Its prevalence differs according to geographic location, age and ethnicity. For example, the prevalence is lower in developing countries, with Malaysia, China, India, Bangladesh, Pakistan, the Philippines, Thailand and Vietnam having a prevalence of <0.5%.^3^ In Malaysia, multi-ethnic groups exist, including Malays, Chinese and Indians, who comprise 68%, 23% and 7% of the national population, respectively.^4^ Malays show the highest prevalence of gout, followed by Chinese and Indians.^5^ Each ethnic group has its own religious beliefs, traditions, festivals and food cultures.

Since gout is a form of arthritis, patients tend to seek a diverse range of complementary medicine, among which dietary supplements and vitamins are frequently used.^6^ Nevertheless, the choice of complementary medicine practices is shown to be influenced by geographical, cultural and social factors, with Asian patients being more likely to use herbal therapy and acupuncture.^6^ In the National Health and Morbidity Survey 2015, the prevalence of traditional medicine practices among Malays, Chinese and Indians was 31%, 33% and 18%, respectively. The types of traditional medicine practised were cupping and massages among Malays, acupuncture among Chinese and Ayurvedic medicine among Indians.^7^

Patients with gout are substantially affected by their symptoms, including not only their mobility during acute gout attacks but also their ability to work and socialise.^8^ As gout is a chronic disease that can present with multiple comorbidities, patients experience a sense of feeling ill that worsens their quality of life.^8,9^ The loss of bodily functions can affect their performance and lead to social isolation, contributing to a progressive loss of self.^9^ Self-management plays an important part in gout management. Adhering to medical management, adapting lifestyle changes for behavioural management and reducing stress for emotional management all contribute to improving gout.^10^

Studies among patients with gout have shown that self-management is influenced by knowledge, cultures and beliefs. Most studies have been conducted in countries with a high prevalence of gout and cultural and sociodemographic profiles different from those in Malaysia. Diet control has been reported to benefit patients with gout relative to their overall outcome. It is known how ethnic cultural beliefs influence the perception and self-management of gout. To date, the overall self-management among patients with gout has not been fully studied. Over the years of the researcher’s practice in managing patients with gout, she has found it difficult to advise patients about their self-management practices, especially those from diflerent ethnic groups, as their cultures, religious beliefs and food habits differ. Therefore, this study aimed to explore the perceptions and practices of short- and longterm self-management of symptoms among patients with gout from diflerent ethnic groups in Malaysia. The findings are expected to help physicians understand patients’ point of view and consequently provide better patient-centred care.

## Methods

### Design

A qualitative study was conducted via semistructured in-depth individual interviews to explore the perceptions and practices of selfmanagement among adult patients with gout.

### Setting, participants, sampling and sample size

Purposive sampling was used to recruit patients who were attending the primary care clinic of University Malaya Medical Centre, were aged more than 21 years, had a confirmed diagnosis of gout by a physician or medical officer based on their symptoms with at least 6 months of duration and were either Malays, Chinese or Indians. Further, patients who were either receiving or not receiving urate-lowering treatments and were able to communicate in either Malay or English language were included. Conversely, patients who were unable to provide consent owing to cognitive impairment were excluded. The sample comprised 20 participants, determined via data saturation, wherein the interviews were concluded when no new themes emerged.

The health belief model was used as the conceptual framework of the study. The topic guide was developed based on the model, which addressed aspects of self-management of gout (perceived susceptibility and severity). The exploration extended to understanding the patients’ lifestyle changes in relation to the cultural practices among their multi-ethnic groups (perceived benefits, barriers and cues to action).

### Data collection

The interviews were conducted by one trained researcher in the preferred language of the participants (either English or Malay), audiorecorded and transcribed verbatim. Field notes, including observed non-verbal cues, were taken during the interviews. Care was taken to minimise potential participant response bias by ensuring that, whenever possible, the participants were interviewed by the researcher’s supervisor.

### Data management and analysis

The data analysis was conducted concurrently with the data collection, reaching completion at the end of a few transcripts. Eight of the interviews were transcribed verbatim by the researcher, and each transcript was checked for accuracy by the researcher’s supervisor. The participants were oflered the opportunity to verify the accuracy of the transcripts, but none of them accepted it. Twelve other interviews were transcribed by an independent transcriber and checked for accuracy by the researcher. Each interview was transcribed using a coding system to ensure anonymity of each participant. The transcripts were not translated, as the researcher was fluent in both Malay and English languages. The transcripts were analysed using QDA Miner Lite software by PROVALIS Research, Canada. The data were analysed using a thematic approach based on Strauss and Corbin’s method involving open, axial and selective coding.^11^ Two of the transcripts were coded independently and compared with the codes obtained by the researcher’s supervisor, and any discrepancy with the codes was discussed until consensus on the list of codes and possible groups of codes was reached to form axial codes.^12^ All remaining transcripts were coded accordingly. The group of codes was later categorised under themes according to the objectives of the study, and irrelevant codes were removed.

## Results

A total of 20 patients participated in the indepth interviews. Among them, 18 were men, and two were women. Three patients refused to be interviewed owing to language barrier (n=1) and lack of interest (n=2). The participants were from the three major ethnic groups in Malaysia: Malays (n=9), Chinese (n=7) and Indians (n=4). The participants’ age ranged from 29 to 81 years. The gout duration ranged from 1 to 30 years. Regarding educational level, the majority of the participants received secondary education, followed by those with a diploma, degree and master’s qualification. One participant received only primary education.

Three themes emerged from the interviews. Together with their subthemes, these themes are summarised in Table 1

**Table 1:** Themes and subthemes.

Themes	Subthemes
Experiences with gout	Help-seeking behaviour
	Causes of gout
	Inevitable pain
	Frequency of gout flares
	Impact of gout
Types of self-management of gout	Diet control
	Usage of pain killers
	Allopurinol medication
	Other types
Factors influencing self-management of gout	Cultural influences
	Other factors

### Experiences with gout

The participants were asked regarding their experiences with gout. In terms of onset, the participants could remember the onset of their gout as being the most painful and severe attack they ever had. Most participants thought that the symptoms were due to a sprain or twist even though they had no previous injury. The majority found that their gout was caused by their poor lifestyle and food habits, especially consumption of foods that can elevate uric acid levels. Some participants also identified that consumption of foods with excess amounts of protein either in the form of meat or vegetables caused their gout. Most participants reported frequent recurrence of acute gout attacks, which were likened to going to war. The participants said that gout substantially affected their quality of life in several ways, including reduction in their mobility and decline in their social and work lives. Regarding the impact of gout on diet, some participants found it difficult to control their diet, as the foods that they were supposed to control or avoid were their favourite. Some participants also experienced worsening of their symptoms over time, including the formation of tophi, causing joint deformities and complications such as renal calculi.

*“Teruk tu maksudnya saya tak boleh berjalan langsung masa tu. Masa tu anak saya bantu saya, saya dipapahlah. ”*

P2, 60, Female, Malay, one year of gout

*“Overall, it’s **like going to war**. Every little while, you get shot here and there. ”*

P15, 71, Male, Malay, 19 years of gout

*“**Quality of life**. Like this gout happened three times when I was overseas you know, so not healthy. You know, when you’re here, when **you have an attack, people said you want to go here, you can’t even join them**. I have been **pushed on a wheelchair at shopping mall so many times** you know. It felt so helpless... You know like being a trouble to people. ”*

P16, 36, Male, Chinese, 14 years of gout

### Types of self-management of gout

Each participant reported varying types of self-management. Diet was the main selfmanagement practice among the participants. The majority of the participants found that managing their diet, especially foods high in purine, could control their gout. Most participants experienced attacks after eating seafood, beef and mutton. Conversely, a few reported that they had attacks after eating vegetables high in protein such as beans, peanuts, pickled vegetables, cabbages, cauliflowers and broccolis. Apart from identifying foods that affected them, the participants also determined which among their favourite foods or drinks they could still consume without limitations. This gradual elimination of foods that triggered reactions was accomplished using the trial-and-error method. Further, some participants discovered that another way to control their gout was to limit the quantity of foods consumed, especially their favourite foods. Apart from diet, usage of pain killers was another main self-management practice reported, especially during acute gout attacks, which are generally substantially painful. Regarding traditional medicine practices, many participants were believers. An example of traditional medicine practised was the consumption of natural products such as celery juice, apple cider vinegar, papaya and pineapple juice. Exercise and stress-reducing activities were among the other types of selfmanagement practices.

*“It’s a very **difficult feeling**.... If the **mutton** is there, mutton is my favourite. I cannot eat... **Prawn** is my favourite, **crab is my favourite**, **cannot eat**, because immediate attack. ”*

P14, 29, Male, Indian, four of years of gout

*“.. Pain killer memang cepat... **Kalau I travel, ada satu pouch. Tapi still, especially travel oversea, I memang bawa la. Standby**. ”*

P7, 43. Male, Malay, 16 years of gout

*“Once I started taking my **pineapple and papaya**, I was able to go back to my normal meal. ”*

P18, 60, Male, Malay, 2 years of gout

### Factors influencing self-management of gout

Malay culture has changed over the years with urbanisation. For example, eating ‘tomyam’, especially seafood tomyam, was reported by the participants to be a practice, especially at urban places. Apart from food, a few Malay participants believed that having gout is a reminder from God not to over eat and to eat healthily. Other participants believed that having gout has no relation to God but is associated with their own doing. When asked about the reasons for not taking allopurinol, some participants mentioned that practising traditional medicine and not relying on modern medicine are part of Malay culture. One participant noticed that he had acute gout attacks after eating ice cream and another participant after drinking iced cube drinks. Accordingly, the relationship of hot and cold foods with gout was explored. The participants believed that eating hot foods was better, as it yielded therapeutic benefits. Although the Chinese participants usually ate hot foods, one participant said that the difference in the temperature of foods triggered no reactions. Among the Indian participants, ‘dhal’ was typically consumed; these participants identified that dhal caused a lot of ‘angin’ (wind), which could be the cause of their gout apart from foods rich in purine. The Indian participants also believed that being overweight would result in lifelong struggle, with no cure for their gout. In addition to cultural factors, family members played major influencing roles, especially those with children or relatives who were healthcare practitioners. Having friends with gout was also another influencing factor reported by the participants. They sought insights from their friends regarding their experiences with gout, comparing notes on factors such as the types of food that aflected them.

*“Most Chinese will take hot food. They like to go for supper, eat mee, “wantan mee”, all hot. I believe that taking hot food has better therapeutic benefits. ”*

P20, 65, Male, Malay, five years of gout

*“Dhal causes pain, maybe because of “angin”. Will eat also, little bit. ”*

P9, 59, Male, Indian, 30 years of gout

*“Well, I **consult with friends** who had this thing and they just recommend to take those medicine la. ”*

P17, 67, Male, Chinese, five years of gout

## Discussion

Contrary to historical descriptions of gout dating back to 2600 BC by the Egyptians, the condition is not viewed as a “rich man’s disease” or ‘disease of kings’.^13,14^ In the present study, the participants reported that although diet was the main contributing factor of gout, there were other causes, such as genetics and medication. According to the literature, gout is seen commonly in patients more than 20 years of age, and its severity increases with age, plateauing by 70 years of age.^1^ Some patients develop gout much earlier at around 16–17 years of age. Gout with an early onset is known as familial gout. This type of gout is recognised as a familial disorder, characterised by a family history of gout. Male members of such families usually develop gout earlier in life, about 7.5 years earlier than those with non-familial gout.^15^

Herein, the participants likened their experience with gout to going to war, facing recurring attacks. The pain was inevitable to all participants and delineated the onset of gout. Their experiences were in accordance to the typical presentation of gout: severe pain waking them up at night or in the morning, commonly at the first metatarsophalangeal joint.^16^ The impact of gout was significant on the patients’ quality of life, social life and work life, consistent with other reports.^17,18^

Diet control was the main self-management practised by most participants. The commonly used ways of diet control were identification of triggering foods via the trial-and-error method and eventual avoidance or limitation of the amount of such foods. The trial-and-error method is an acceptable way of identifying which food affects patients, as each of them reacts differently to different types of food. Evidence shows that certain diet and amount consumed affect the clearance of uric acid from the kidneys.^19^ In this study, the participants reported being empowered by the decision of taking medication, as advised by their physicians. Regarding traditional medicine practices, there were believers and non-believers among the participants. The reasons for practising traditional medicine were to search for ways to control or cure their gout to avoid dependence on modern medications. Eating pineapple and papaya was found to improve their gout symptoms compared with the other practices used. This finding is consistent with a report showing that the leaf extracts of papaya yielded xanthine oxidase-inhibitory effects compared with other parts of papaya.^20^

Among Malay groups, certain food cultural practices have changed over the years owing to urbanisation. Most Malay foods at stalls and restaurants are hybrid forms influenced by Thai, Chinese and French cuisines to suit the taste of locals and foreigners.^21,22^ Among Chinese groups, food is used to establish relationships among people, such as making new friends or business partners. For example, their dinners usually consist of 4–10 dishes depending on the occasion and are shared together in a round table to unite people together.^23,24^ Among Indian groups, eating dhal is part of their staple food with rice. In the present study, consumption of ‘chapati’ or ‘thosai’ was reported to cause acute gout attacks among the Indian participants. Some participants were even advised to avoid eating dhal, as it causes wind and can aggravate their gout symptoms. This finding is in contrast to a report showing that dhal has anti-inflammatory effects, and as a legume, this vegetable protein has less effect on gout than animal protein.^25^ Patients with diseases that are considered cold, such as arthritis, rheumatic arthritis and neuralgia, are advised to restrict foods that are also considered cold.^26^ Cold foods such as leafy vegetables and most fruits have higher water content and lower fat, protein and carbohydrate contents.^27^ Herein, some participants reported that cold foods including ice cubes and ice cream triggered attacks. Nevertheless, further studies are needed to delve into the association of cold foods with gout attacks.

One strength of this study is the use of in-depth interviews; this approach allowed the researcher to explore the participants’ perceptions and cultural practices of self-management of their gout. Another strength of this study is that the participants had different demographic profiles, allowing the exploration of a wider range of experiences of self-management practices. In contrast, the limitation of this study is that it was conducted at a primary care clinic in an urban setting, making it difficult to explore cultural practices in greater detail. Nevertheless, the findings suggest that patients can be advised by healthcare practitioners about using a food diary, where they can individualise their own diet using the trial-and-error method. Further research must explore the facilitators and barriers to counselling by healthcare practitioners in primary care settings relative to self-management among patients with gout.

In conclusion, diet control is the main selfmanagement practice of patients with gout. Traditional medicine practices include the use of natural methods. Each ethnicity has its own unique beliefs and food cultures. Therefore, by understanding the self-management practices of patients from different ethnicities, healthcare practitioners can personalise the management of gout.

## Data sharing statement

The data that support the findings of this study are available on request from the corresponding author. These data are not publicly available since they contain information that could compromise the privacy of the participants.

**How does this paper make a difference in general practice?**

1. This paper can benefit those practicing in the field of traditional medicine.
2. This paper would help future researchers in their literature review for future research.
3. This paper could help future researcher in coming out with ideas of future research in the field of gout.
4. This paper can also be a guide to dietitian in improving their consultations to patients with gout who are referred to them.
5. This paper can be a guide to healthcare practitioners in improving the self-management of patients with gout in primary care.
